# Chicago Medical Society

**Published:** 1885-04

**Authors:** 


### SOCIETY PROCEEDINGS.

TRANSACTIONS OF THE CHICAGO MEDICAL SOCIETY.

*Regular Meeting, Jan. 19th, 1885.*—DR. E. J. Doering, President, *pro tem.;* Dr. Liston H. Montgomery, Secretary.

*The Bacillus of Syphilis, and the Treatment of the Disease with Tannate of Mercury.*—In microscopical sections from two syphilitic chancres and a syphiloma, Lustgarten had found bacilli which were perfectly characterized by their color reaction, their form, and their relative position. They were represented as little rods, straight or somewhat curved, slightly larger than the bacilli of tuberculosis, but of the same general appearance. They were found single or in small groups, inclosed in somewhat distended lymphoid cells, and, with powerful illumination, showed spots like those that Koch regarded as spores in the tubercle bacilli. Their staining properties made it possible to distinguish them from all other pathogenetic organisms, and the fact of their being always inclosed in cells would prevent their being confounded with putrefaction formations, etc. As the author alluded to had cultivated this bacillus, and as his results had been confirmed by Koch, it would seem that the evidence was sufficient.

In regard to the treatment of the disease with the tannate of mercury, the first trials had been made by Kaposi, nearly a year ago, and the results had been so remarkable, and the advantages so great, that the drug was now freely used in Germany. It was a tannate of the protoxide, was without odor or taste, and was soluble only when decomposed. It contained about fifty per cent. of metallic mercury. Hydrochloric acid made no sensible impression on it; it was easily dissolved in nitric acid; very dilute alkalies, such as solutions of ammonia, caustic potash, or an alkaline carbonate, reduced it to a sort of magma, composed of very small particles of metallic mercury. Its rapid absorption into the system was proved by an abundant mercurial precipitate found in the urine shortly after it was administered. It was given in nearly all forms of syphilis—recent as well as inveterate eruptions and gummy deposits. Dr. Zeisler had himself seen the patients, whose cases had been reported in one of the Vienna journals, and could testify to the results. Six of the cases were primary eruptions, three were gummata, two showed small papules and large pustules, and one was a case of relapsing secondary syphilis. A rapid diminution of the lesions would be observed in the course of a week or two, and in three or four weeks they would have entirely disappeared.

The author then alluded to a case that he had under treatment lately, that of a man who had contracted a chancre last June, at which time he received treatment by another physician. In six weeks the roseola appeared, together with various other manifestations. A number of remedies were used internally, and external treatment was employed, until September 14th, when the patient first consulted him. At that time there was a widely diffused maculo-papular syphilide. As it was impossible to obtain any of the tannate of mercury then, mercurial frictions were used until September 19th, but from this latter date until October 5th, the patient took four grains and a half of the tannate daily. The eruption had then entirely disappeared, and only a few spots of pigmentation could be seen; there was no sign of salivation. The use of the drug was continued until the 1st of November, and the patient was then discharged cured.

The remedy was usually given in doses of about a grain and a half, with four or five grains of sugar, three times a day, an hour after meals. When thus given, it was rare for it to produce any sign of salivation. The alkaline carbonates, and considerable quantities of mineral waters containing such salts, should be avoided by patients, who were taking the remedy, for they decomposed it; and iodide of potassium should not be given at the same time, as the production of a large amount of iodide of mercury would be the result. The writer had never seen any disagreeable symptoms of mercurial poisoning produced while the drug was being used, nor any irritation of the bowels, even when its use was continued for weeks in such large doses as six grains daily, and he had seen about twenty cases so treated. If the bowels became loose, one sixth of a grain of opium could be added to each dose. While this new remedy might not produce a revolution in the treatment of syphilis, it deserved to be classed among the best that had come into use.

Dr. G. C. Paoli asked with regard to the use of the drug in the tertitiary form, where the bones were involved, and stated that its employment had been suggested as early as six years ago in some of the foreign journals. The main remedies that he relied upon were iodoform for chancres, and afterward fumigations and the use of the red iodide of mercury in small doses, and he had yet to see his first case of salivation from that treatment, which he could not say of the green iodide. He sometimes found it necessary to use a little opium or hyoscyamus, to prevent irritation of the bowels. The tannate might have astringent properties, and, if it had, it might be very good for children. Where the bones were affected, he thought iodide of potassium, in doses of fifteen, twenty, or thirty grains, was unequalled as a remedy, but the disease was one in which we could not say that any remedy was the best, for there were special idiosyncrasies in some cases. Regarding the micro-organisms of syphilis, Dr. Saunders had discovered them ten years ago, but he had been ridiculed for his ideas, and had desisted from further investigations in htat direction.

Dr. Robert Randolph had seen pathological specimens from syphilitic persons. He prepared slides, purporting to contain bacilli, at least three years ago, but it had then been concluded that there was nothing in the supposed discovery.

Dr. Zeisler thought that Dr. Paoli must be mistaken about the alleged discovery of syphilitic bacilli ten years ago, for certainly nothing was known about coloring or cultivating bacilli until within the past few years.

*A New Method of Counting a Rapid Pulse* was the title of a short paper by Dr. A. E. Hoadley. The method consisted simply in counting every alternate pulsation and multiplying the number by two. On the first trial one would probably fail to make an accurate count, but with a little practice the knack was soon acquired, and the acquisition might be hastened by practicing on the tick of a watch.

*Obstruction of the Ileum by Peritoneal Adhesions; Death.*—Dr. Hoadley also reported the following case:

A man, thirty years old, apparently in good health, was attacked very suddenly, in the night, March 14th, with severe pain in the abdomen, opposite the umbilicus. It continued for five or six hours, from the effect of remedies, as was supposed. After a few hours it returned and again subsided. With some variation in the frequency and severity of the paroxysms, it continued up to the time of his death, four weeks from the time of the seizure. Soon after the onset he began to suffer with vomiting of a very distressing nature, but in time this became more passive, although it continued from day to day to the last. At times he vomited simply what he had swallowed, but at other times, and especially during the first two weeks, the vomiting was stercoraceous. For three weeks the temperature did not exceed 100° F., and most of the time it was about normal, while the pulse ranged between 75 and 85, but gradually grew weaker, and during the last few days was very rapid and scarcely perceptible at the wrist. There was no tympanites, and but very little tenderness. Much of the time the abdomen was quite flat and had a doughy feel, but during the paroxysms it became as hard as a contracting uterus during labor, although there was no muscular rigidity or increase of tenderness. During the intervals gentle kneading would reproduce the tense condition without producing pain. This state of things was most marked during the last week of the illness, at which time the patient was much exhausted, with his voice husky, his skin of a leaden color, and presenting a general pinched and emaciated appearance. The pains had then lost their severity, a sense of suffocation with general numbness being produced instead—as he described it, it felt as if his stomach was being crowded up under his ribs. Throughout the case, except on one day, there was no voluntary passage from the bowels, although before the attack they had been soluble and very regular. With the aid of injections and laxatives, a few small, elongated lumps of fæcal matter, never exceeding three-eighths of an inch in diameter, were passed every day or two. At the end of the third week, however, there was a slight diarrhœa for one day only. Throughout his entire sickness, even in the absence of pain, he was restless and weary, and never slept more than an hour at a time.

Treatment was of little avail. Opium and chloroform were the principal remedies used; they retarded the vomiting and pain, but controlled neither. Many other remedies were tried, and the case was treated from every possible standpoint, except that of surgical interference; there seemed to be no time when an operation was admissible. Rectal alimentation appeared to give good results. Dr. J. D. Skeer and Dr. C. W. Earle were called in consultation, and confirmed the writer's early diagnosis of mechanical obstruction of the small intestine, probably at the ileo-cæcal junction. The patient died on the twenty-eighth day. The autopsy revealed many peritonieal adhesions and bands of long standing, which were very firm, binding the ileum down into the pelvic cavity. One of them was firmly attached to the anterior abdominal wall, midway between the umbilicus and the middle of Poupart's ligament on the right side. At its attachment it was about one-fourth of an inch thick and three-fourths of an inch wide. It then spread downward to the left side of the pelvic cavity, where it was firmly adherent, from the brim far down upon the inner wall, becoming fasciculated and embracing the folds of the ileum between the fasciculi, furnishing a band that held the larger mass of the small intestine in the pelvis. On dividing these bands and removing the intestines, they were found everywhere bound together by adhesions; but there was not the slightest evidence of recent inflammation of the peritonæum, although the intestine itself was inflamed and very much softened for a distance of about four feet, extending to within twelve or fourteen inches of the ileo-cæcal junction. There was no evidence of previous disease of the intestine.

Eight years before the patient had been treated by the writer for typhoid fever, which ran a moderately severe course, and ended in recovery. A most interesting point about the case, as it now appeared, was the fact that at times he had a moderate peritonitis, which in all probability had given rise to the adhesions.

Dr. A. Leigh would like to know how the tense condition of the abdomen took place without muscular contraction.

Dr. F. Carey asked if there were any adhesions below the ileo-cæcal valve.

Dr. Hoadley replied that the valve was normal. So, too, was the caliber of the whole length of the colon; the small size of the passages was in part due to the pelvis being full of intestine, so that the fæces were molded, as it were. Regarding the tense condition, there was a spasmodic contraction of the bowels, which was uninfluenced by the will.

